# Caustic recovery from caustic-containing polyethylene terephthalate (PET) washing wastewater generated during the recycling of plastic bottles

**DOI:** 10.1038/s41598-025-85365-9

**Published:** 2025-01-23

**Authors:** Aya Alterkaoui, Ozan Eskikaya, Bulent Keskinler, Nadir Dizge, Deepanraj Balakrishnan, Pavan Hiremath, Nithesh Naik

**Affiliations:** 1 Department of Environmental Engineering, Mersin University, 33343 Mersin, Turkey; 2 Department of Energy Systems Engineering, Tarsus University, 33400 Tarsus, Turkey; 3 Department of Environmental Engineering, Gebze Technical University, 41400 Gebze, Kocaeli Turkey; 4 Department of Mechanical Engineering, Prince Mohammad Bin Fahd University, 31952 Al-Khobar, Saudi Arabia; 5 Department of Mechanical and Industrial Engineering, Manipal Institute of Technology, Manipal Academy of Higher Education, Manipal, Karnataka 576104 India

**Keywords:** PET washing wastewater, Coagulation-flocculation, Ultrafiltration, Nanofiltration, NaOH recovery, Wastewater recycling, Engineering, Materials science, Nanoscience and technology

## Abstract

To prevent water scarcity, wastewater must be discharged to the surface or groundwater after being treated. Another method is to reuse wastewater in some areas after treatment and evaluate it as much as possible. In this study, it is aimed to recover and reuse the caustic (sodium hydroxide, NaOH) used in the recycling of plastic bottles from polyethylene terephthalate (PET) washing wastewater. Chemical substances used in the industry will be significantly reduced with chemical recovery from wastewater. Ultrafiltration (UP150) and nanofiltration (NP010 and NP030) membranes were used for this purpose in our study. Before using nanofiltration membranes, pre-treatment was performed with coagulation-flocculation process to reduce the pollutant accumulation on the membranes. Different coagulants and flocculants were used to find suitable coagulants and flocculants in pre-treatment. The pre-treated wastewater using aluminum oxide, which supplied the highest chemical oxygen demand (COD) removal (76.0%), was used in a dead-end filtration system to be filtered through NP010 and NP030 membranes at different pressures (10–30 bar). In the same filtration system, raw wastewater was filtered through a UP150 membrane. Among these treatment scenarios, the best method that could remove pollutants and provide NaOH recovery was selected. After each treatment, pH, conductivity, COD, and NaOH analyses were performed. The maximum NaOH recovery (98.6%) was obtained with the UP150 membrane at 5 bar.

## Introduction

Wastewater is released into many areas as a result of industrial production. Pollutants that cannot be easily degraded, such as organic-inorganic substances, volatile organic compounds, heavy metals, and various chemicals, may be found in wastewater^1^. The contents of industrial wastewater may vary depending on the materials and units used and the operating conditions^2^. Pollution of water with chemicals containing acidic or alkaline compounds changes the pH of clean water, which is close to neutral. Water pipes are exposed to acidic contamination in both rust and corrosion^3^. When exposed to alkaline-based pollutants, the hardness of water increases, which also leads to increase salinity in the discharged soil. Alkali pollution is generally caused by carbonates, chlorides, and hydroxides^4^. The main sources of alkaline wastewater in industrial areas include industries in which caustic soda is frequently used, such as chemical fibers, leather, and caustic soda^5^. Sodium hydroxide (NaOH), also known as caustic, is the most common type of wastewater used in various industrial areas. Water polluted with NaOH or caustic has a pH value higher than 12 and a salinity of 5.8–7.5% by weight. These values pose a threat that can seriously affect the health of living organisms and negatively affect their ecology^6^.

It has become important not only to purify all wastewater used for different purposes but also to ensure its reuse. Today, resource scarcity due to the increasing population requires the recycling of the remaining materials and resources after use^7^. During wastewater recycling, both water needs can be met, and valuable compounds can be recovered and reused as a resource^8^. The main purpose of wastewater treatment is to reduce environmental damage and protect existing clean water resources. The direct discharge of water containing pollutants may render groundwater and surface water unusable. Currently, chemical, thermal, biological, and membrane treatment technologies are frequently used to treat wastewater^9,10^. Biological treatment systems are practical and cost-effective in terms of their operating conditions. However, the difficulty of ensuring biological activation in wastewater with high pH values can be partially eliminated in alkaline wastewater, and high pH conditions (pH 7–8) suitable for the living conditions of microorganisms must be eliminated^11^. One of the chemical treatment procedures is coagulation-flocculation, which is based on the fact that small particles interact with the coagulant to form large flocs, and the formed flocs collapse due to gravity. Suspended solids, organic matter, heavy metals, and colors can easily be removed from water using this simple procedure^12^. In addition, membrane filtration systems can provide high-quality purified water with different pore sizes (ultrafiltration, nanofiltration, and reverse osmosis). Chemical treatment processes can be used in variable operating conditions such as high pH values and they are more resistant to sudden changes in wastewater content. Chemical treatment processes are not affected by higher hydraulic loadings compared to biological treatment systems. They provide high efficiency in removing suspended solids and even micropollutants. In addition, pretreatment processes with chemical treatment are frequently used to prevent clogging problems of membranes. Therefore, using them for pretreatment purposes in membrane treatment systems significantly reduces the pollutant loading on the membranes^13,14^. Membrane technology is widely used in the textile industry for wastewater treatment and caustic detection. Ultrafiltration (UF) is a membrane filtration process commonly used to recover particles and macromolecules from fabric residues. Additional techniques, such as nanofiltration (NF) and reverse osmosis (RO), are required to separate the biological components of low-molecular-weight and divalent salts, resulting in caustic recovery^15^. Nanofiltration (NF) membranes have a pore size of ~ 1 nm, thus rejecting more solutes than ultrafiltration membranes, and have higher permeability than reverse osmosis membranes^16^. NF membranes can separate many contaminants from water and allow compounds, such as NaOH, to be recycled in the resulting filtrate by passing them through their pores^17^. Considering all treatment systems, the use of only one type of treatment system may be insufficient for the intended use of treated water. Therefore, the combined use of several different treatment systems can increase the quality of the treated water and increase the lifespan of the treatment systems^18^. If high concentrations of contaminants pass directly through the membrane, the pores will become clogged in a short time, and the membrane will either have to be replaced or the pores will have to be cleaned^19^. It would be appropriate to pre-treat wastewater to extend the life of the membrane and prevent high pollution^20^.

The reuse of treated water for its intended use is possible when the treatment procedures employed for purification are performed properly. Considering that the water demand needed in 2030 will be 6900 billion m^3^, reusing water is another method with increasing water demand. The reuse of refined or partially purified water in processes deemed suitable for industrial use has also gained great importance. Water reuse activities have increased exponentially in the last decade^21^. The recycling facility for polyethylene terephthalate (PET) skewers is also effective in the formation of caustic wastewater used in many areas. The most important step in recycling PET bottles is to thoroughly clean the waste and prevent the presence of harmful pollutants. The water released during washing has mixed components and contains high concentrations of pollutants (such as organic matter, COD, pH, and conductivity)^22^. PET-added materials are frequently used in every aspect of our daily lives. An average of 3.48 kg of water per kilogram is used in recycling centers to wash these PETs and prepare them for reuse. With increasing population, the amount of washing solution needed to recycle these PET materials will also increase. After cleaning these waters, producing and reusing caustic-containing water may also be important for preventing waste^23^.

There are very few studies on caustic recovery from PET washing wastewater. In this study, it was aimed to determine the best recovery system to prevent caustic recovery and NaOH loss from caustic-containing wastewater. The pollutant concentration of the real wastewater was reduced using the coagulation-flocculation process before the membrane treatment process. The pre-treated water obtained after coagulation-flocculation was then used with two NF membranes of different pore sizes (NP010 and NP030) under three different pressures in the dead-end filtration system. In addition, wastewater containing raw caustic was passed directly through an ultrafiltration membrane (UP150) without pre-treatment. Thus, we aimed to determine the most suitable treatment conditions for caustic recovery by using two treatment systems. To evaluate the results, chemical oxygen demand (COD), pH, conductivity, and NaOH analyses were performed.

## Materials and methods

### Coagulation-flocculation pre-treatment of caustic-rich wastewater

Jar test experiments (Mtops Four Jar Test Device, SF4 Model) were performed at the original pH value of the wastewater. 250 mL of wastewater sample was placed in 600 mL beakers and mixed at 150 rpm for 5 min for coagulation (fast mixing). Then, the mixing speed was reduced to 50 rpm for 20 min for flocculation (slow mixing). The flocs formed in the beakers were allowed to settle for 30 min. COD analyses were performed in the upper phase wastewater and the most suitable coagulant was selected to use in the membrane system. In this study, five chemical coagulants, aluminum sulfate (Al_2_(SO_4_)_3_ × 18H_2_O, Merck, ≥ 97%), iron(III) chloride (FeCl_3_, Tekkim, 98%), iron(II) sulfate heptahydrate (Fe_2_SO_4_ × 7H_2_O, Merck, 99.5%), polyaluminum chloride (PAC, Tegetkimya, 17% Al_2_O_3_), and aluminum oxide (Al_2_O_3_), were used. Then, the obtained results were compared. The optimum conditions of the chemical coagulant Al_2_O_3_, which achieved the best COD removal efficiency, and the coagulant concentration with flocculant were determined and wastewater was collected under optimum conditions. Then, collected pre-treated wastewater was used in the dead end filtration system using ultrafiltration (UF) and nanofiltration (NF) membranes.

### Caustic recovery using membrane filtration after coagulation-flocculation pre-treatment

**Fig. 1:**
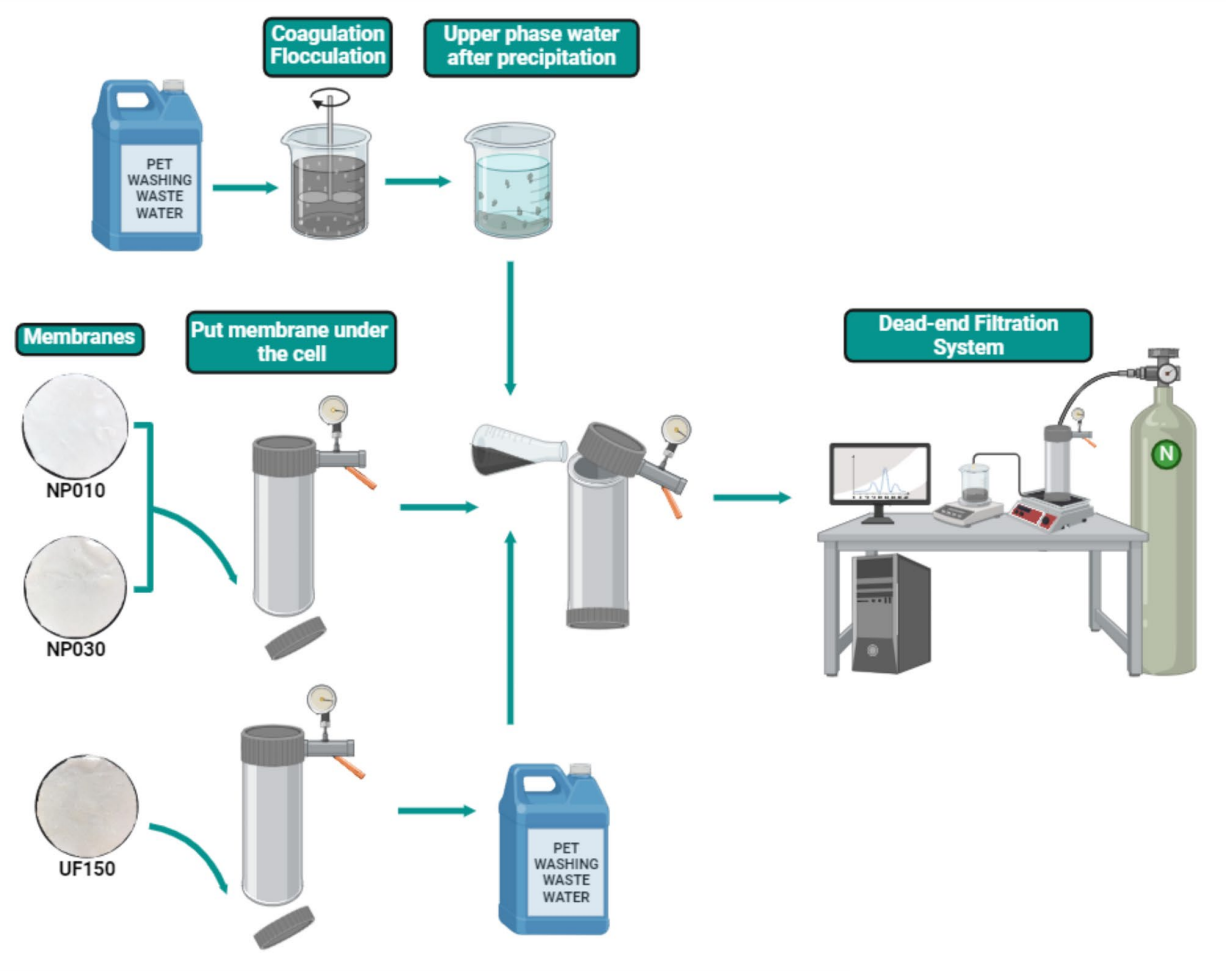
Use of raw and pre-treated wastewater in dead-end filtration system with different membranes.

Membrane filtration studies were carried out using a dead-end filtration system (Sterlitech HP4750 Stirred Cell) (Fig. 1). A manometer was used to apply the required pressure using inert nitrogen gas. The filtrate flow was monitored by automatically weighing every minute using the RS-COM program. The flux values of the membranes were calculated using Eq. 1. Three different membranes (UP150, NP010, and NP030) were used in the filtration experiments. Details about the membranes are given in Table 1. The effective membrane area was 14.6 cm^2^. These membranes were chosen for this study because they can easily pass NaOH and are easy to obtain. The molecular weight cutoff value is 1000 Da and 500 Da for NP010 and NP030 membranes^24,25^, respectively, and 150 kDa for the UP150 membrane^24^. When the pure water flow values of these two different nanofiltration membranes were compared, the values were 100 L/h.m^2^ for the NP010 membrane and 40 L/h.m^2^ for NP030. In direct proportion to this situation, the water permeability of NP010 is higher than that of NP030. The maximum pressure at which both polyethersulfone membranes could operate up to 40 bar. The NP010 and NP030 membranes were studied at pressures of 10, 20, and 30 bar with upper phase wastewater after precipitation, and the UP150 membrane was studied at pressures of 5, 7.5, and 10 bar with raw wastewater.

1 $$\:Flux\:\left(L/{m}^{2}h\right)=\:\frac{{\Delta\:}\text{V}}{{A}_{X}.{{\Delta\:}}_{t}}$$

where ΔV is the amount of permeate sample collected over a given period of time (Δ_t_, h) (L) and A is the membrane area used for filtration (m^2^).

**Table 1:** General properties of the membranes used in the study.

Membrane	Material	Max. temperature (°C)	pH	Molecular weight cutoff (MWCO)
UP150	Polyethersulfone (PES)	95	0–14	∼150,000 Da
NP010	Polyethersulfone (PES)	95	0–14	∼1000 Da
NP030	Polyethersulfone (PES)	95	0–14	∼400–600 Da

Pre-treated caustic-containing wastewater and raw wastewater were added to the dead-end filtration system at a volume of 250 mL, and water was allowed to pass through for 4 h. During the filtration experiments, care was taken to collect the flux such that no more than 70% of the total volume was filtered. COD, pH, conductivity, and NaOH analyses were performed for each filtrate sample that passed through the filtration system. All our experiments were performed in triplicate.

### Analytical methods

A digital pH and conductivity meter (WTW Multi 340i Model) was used for pH and conductivity measurements. The COD analysis of the samples was carried out in accordance with the titrimetric method (Standard Methods 5220 B)^26^. The results of the COD analyses were evaluated by calculation according to Eq. 2. NaOH determination was based on the study by Harvey^27^. A titration solution containing 1 N hydrochloric acid was prepared. It was prepared by dissolving phenolphthalein (1 N) in ethanol. Pure water (50 mL) and caustic samples (2 g) were added to a 100 mL flask. 2–3 drops of phenolphthalein solution were added. The titration solution was added dropwise to the sample using a graduated burette with constant stirring. The sample was titrated until its color changed from pink to colorless, and consumption was recorded. The amount of NaOH in the sample was calculated as a percentage, using Eq. 3.

2 $$\:COD\:Removal\:\left(\%\right)=\:\frac{{C}_{0}-\:{C}_{e}}{{C}_{0}}\:x\:100$$

3 $$\:NaOH\:\left(\%\right)=\:\frac{{V}_{t}\:x\:4}{{V}_{s}}$$

where C_o_ is the concentration before treatment, C_e_ is the concentration after treatment, V_t_ is the amount of titration solution added until color change is observed, and V_s_ is the sample amount.

## Results and discussion

### Chemical coagulation-flocculation pre-treatment results

**Fig. 2:**
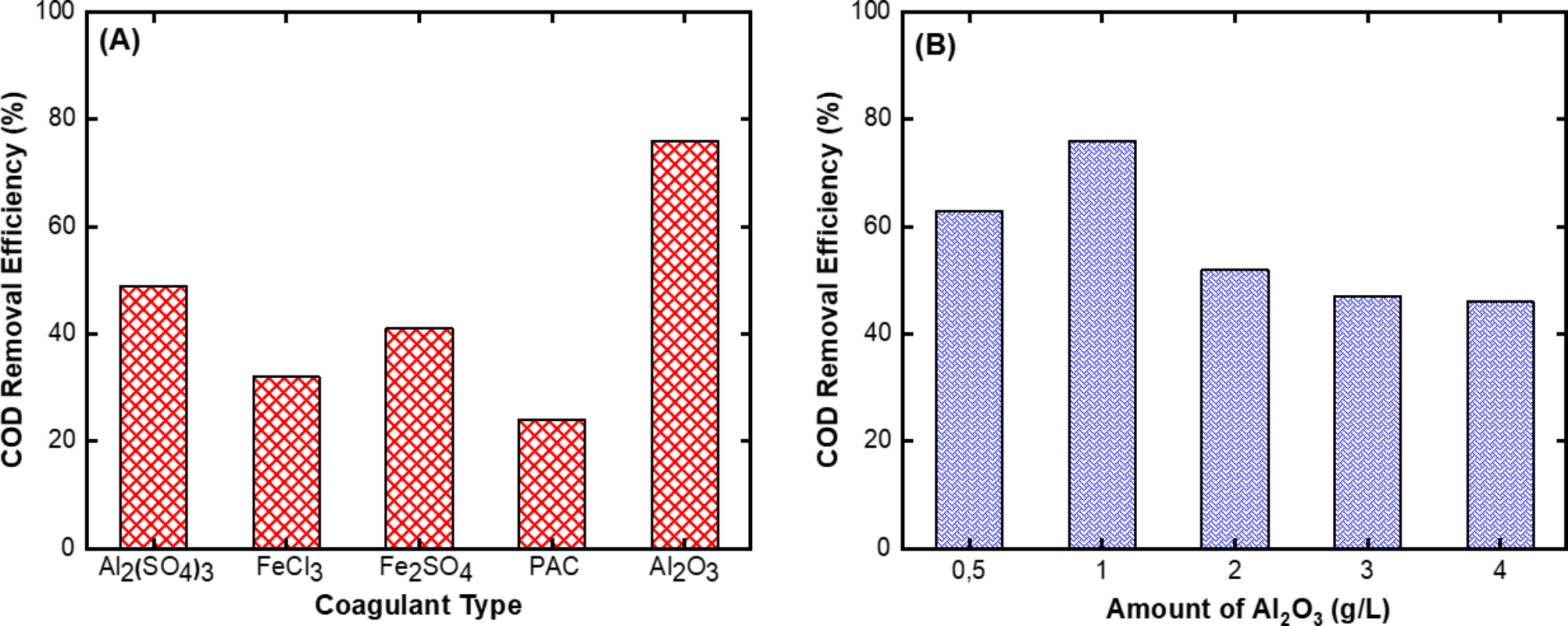
Optimization results on COD removal efficiency for pre-treatment of wastewater containing caustic by coagulation-flocculation; **(A)** the effect of coagulant type and **(B)** the effect of Al_2_O_3_ amount.

The purpose of pre-treatment is to prevent the rapid clogging of NF membranes. The COD removal efficiencies were used to determine the most suitable coagulant and flocculant for wastewater containing caustics (Fig. 2). In preliminary studies, the use of 1 g/L Al_2_(SO_4_)_3_, FeCl_3_, Fe_2_SO_4_, PAC, and Al_2_O_3_ coagulants and 5 mL/L cationic flocculant (CF, 0.1% w/v) was chosen because better sludge settling was observed. The same situation was used with 5 mL/L of anionic flocculant (AF, 0.1% w/v), as it was more effective in Al_2_O_3_. After each experimental set and a 30 min holding period, the water in the upper phase was collected, and COD analyses were performed. In coagulation-flocculation experiments using CF along with Al_2_(SO_4_)_3_, FeCl_3_, Fe_2_SO_4_, and PAC, the COD removal efficiencies were determined as 49%, 32%, 41%, and 24%, respectively (Fig. 2A). Keeping the amount of 5 mL/L of AF constant, the amount of coagulant for Al_2_O_3_ was added to the wastewater containing caustic between 0.5 and 4.0 g/L. The best COD removal efficiency (76%) for this coagulant was obtained in the experimental set with 1 g/L of Al_2_O_3_. In the experiment using 0.5 g/L Al_2_O_3_, the COD removal efficiency was calculated as 63%. In addition, in the experiments where 2 g/L (52%), 3 g/L (47%), and 4 g/L (46%) Al_2_O_3_ were used, the COD removal efficiency started to decrease with increasing amounts of coagulant (Fig. 2B).

The presence of more coagulants in wastewater than the optimum conditions is considered an additional source of COD in wastewater^28^. Because coagulants are used to remove pollutants that can be removed by coagulation in water, the excess will remain free in the water as it does not react and will increase the COD concentration^29^. Tlaiaa et al.^30^ reported a similar result in their study. In experiments where they used FeSO_4_ to remove Reactive Black 5 dye, they observed that the COD removal efficiency decreased at the highest dosage, while determining the optimum coagulant amount. In addition, the presence of excessive amounts of coagulant in pollutant-containing water may cause a situation that will prevent the formation of flocs and thus COD removal efficiency may decrease. Boguniewicz-Zablocka et al.^28^ also explain this situation. In their study, coagulation treatment was applied to purify paper industrial wastewater. They explained the importance of determining the amount of coagulant by the release of sludge formed when excessive amounts of coagulant caused deflocculation. According to the results obtained for the caustic water used in our study, it was determined that the anionic flocculant with 1 g/L of Al_2_O_3_ had the best COD removal efficiency. For this reason, the upper phase liquid sample obtained from the sample subjected to large volumes of coagulation-flocculation treatment was collected and stored for use in the membrane filtration system.

### Membrane filtration results

The cell, which had a membrane at the bottom of the dead-end filtration system, had a volume of 250 mL. The wastewater to be filtered was added, filtered through the membrane with the pressure obtained by the nitrogen gas from above, and the resulting filtrate was collected in a chamber at the cell exit. To obtain permeate water, NP010 and NP030 membranes were operated under 10, 20, and 30 bar pressure after pre-treatment, and UP150 was operated under 5, 7.5, and 10 bar with raw caustic wastewater. COD, pH, conductivity, and NaOH analyses were performed in the filtrates. Since the aim of our study is to obtain caustic recovery, it is necessary to select the membrane and operating conditions that have the least NaOH rejection after pre-treatment and are beneficial in terms of COD removal.

#### Flux data of the UF and NF membranes

**Fig. 3:**
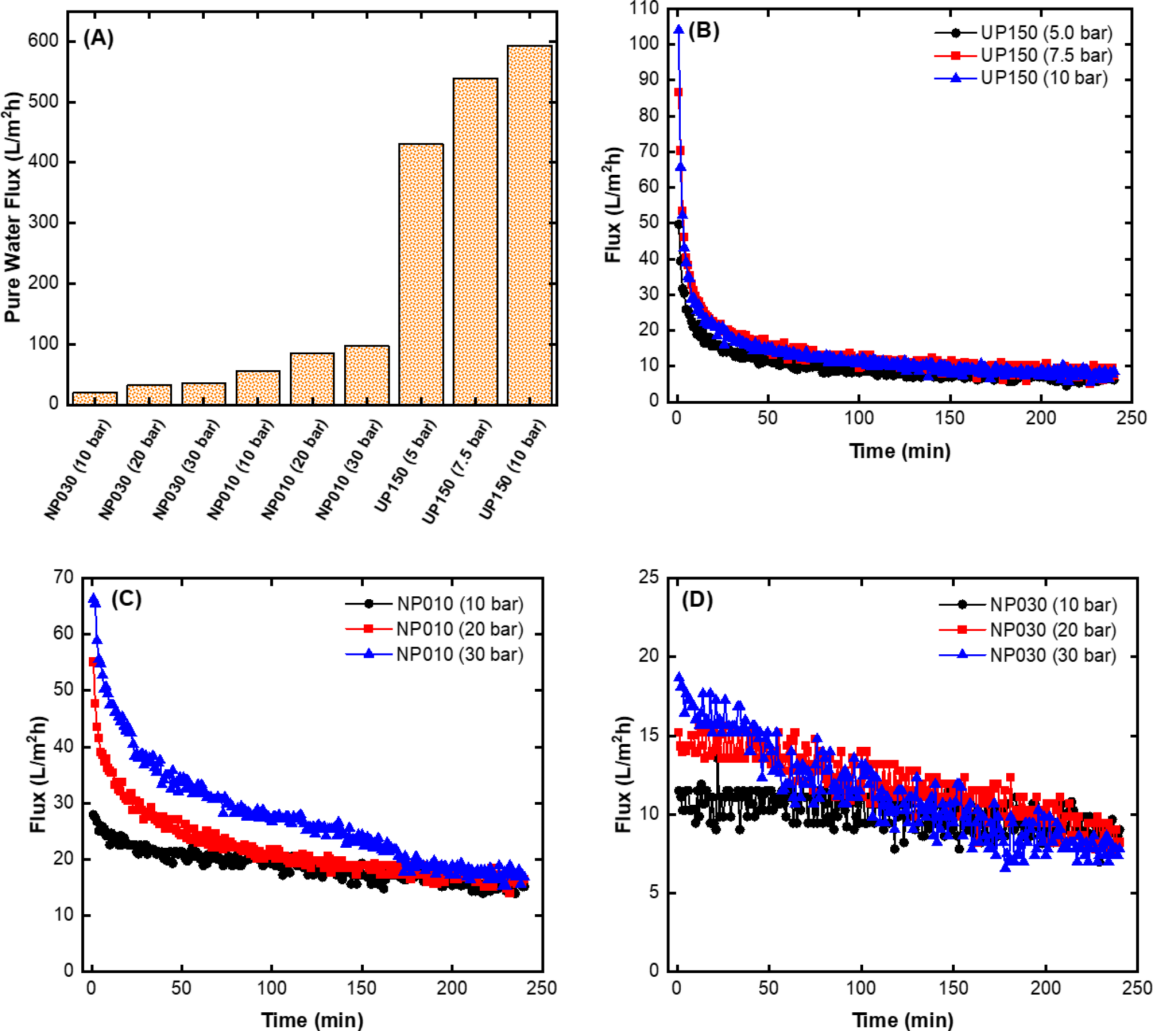
Flux graphs of UF and NF membranes; **(A)** Pure water fluxes, **(B)** Real wastewater fluxes of UR150 membrane, **(C)** Real wastewater fluxes of NP010 membrane, and **(D)** Real wastewater fluxes of NP030 membrane.

After the membranes are placed in the dead-end filtration system, it is important to monitor the pure water fluxes under certain pressures to fully open the pores of the membranes and place the membrane on the filtration base^31^. Unlike fluxes of pure water, fluxes are expected to gradually decrease versus time due to blockage by contaminants that cannot pass through the pores during real wastewater flowing through the membrane^32^. All membranes used in this study were placed in a dead-end filtration system, and then pure water was allowed to pass under certain pressures. After the pure water fluxes reached a constant level, the dead-end filtration system was emptied, caustic rich wastewater was added to the system, and the fluxes versus time were automatically recorded (Fig. 3). Upon examination of pure water fluxes, it was found that pure water fluxes increased with an increase in both pore size and pressure. This is an expected situation, considering the operating conditions of the membranes (Fig. 3A). For NP030 < NP010 < UP150, the pore structure is such that an increase in pore size corresponds to a decrease in the amount of pure water that passes over time. Shakiba et al.^33^ synthesized a polyacrylonitrile/PANI composite membrane for the development of superhydrophilic nanofiber membranes. In their study, they observed that a larger pore size caused an increase in the flux in the membrane, similar to our study. The UP150 (Fig. 3B), NP010 (Fig. 3C), and NP030 (Fig. 3D) membrane fluxes decreased over time. This can be explained by the fact that the membrane pores became clogged by pollutants over time, reducing the amount of water allowed to pass. Kadhim et al.^34^ used graphene oxide mixed matrix membranes and attributed the decrease in permeate flow to pore clogging caused by a high graphene oxide concentration in their study.

#### COD removal performances of the UF and NF membranes

**Fig. 4:**
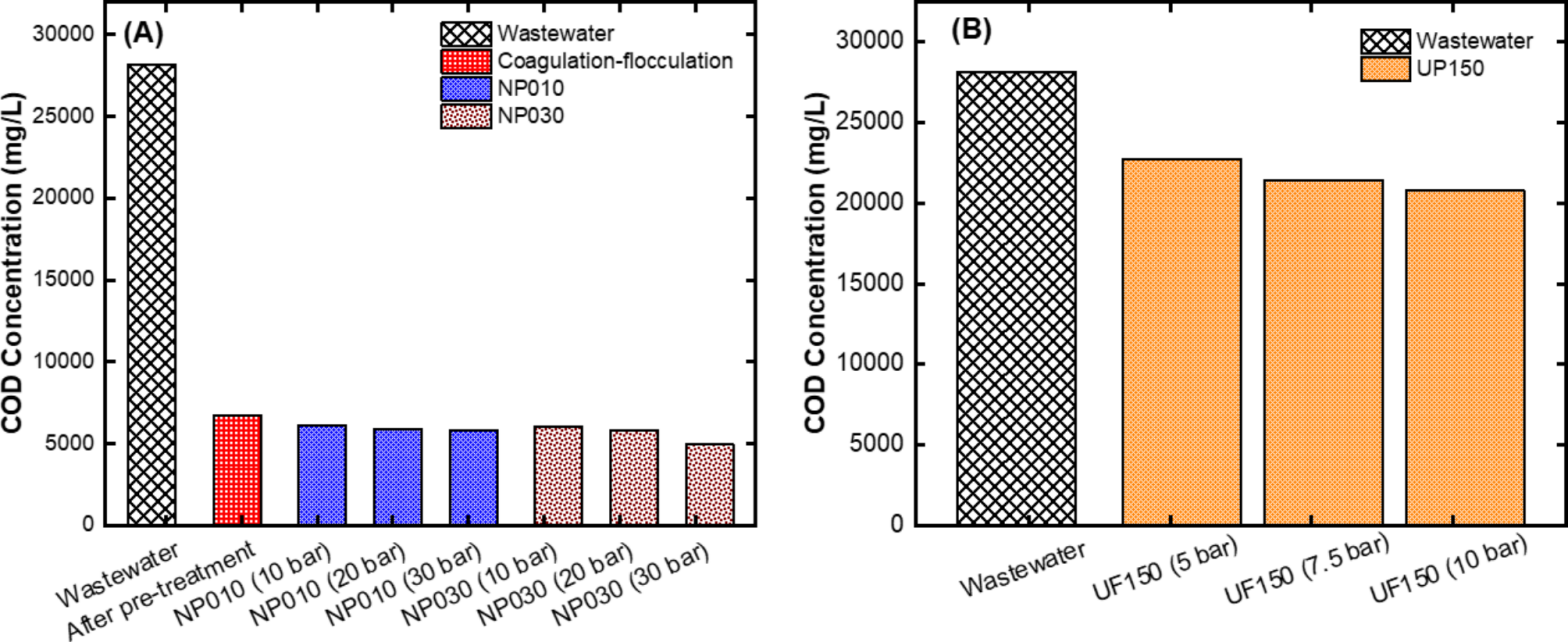
Results of COD removal for **(A)** NF membranes after pre-treatment with coagulation-flocculation process and **(B)** UF membrane without pre-treatment.

Depending on the intended use of the plastic materials, many contaminants may be present on the inner and outer walls. There may be high concentrations of compounds, especially cleaning chemicals, such as detergents, oils, organic matter, and suspended solids, which can cause turbidity. After washing, all these impurities pass into the water, and it is inevitable that the wastewater will have a high COD concentration^35^. Therefore, in our study, analysis was performed for each sample after treatment to determine the status of the COD sources in the caustic-containing water during its recovery (Fig. 4). The COD concentration of the real caustic rich wastewater was approximately 28,000 mg/L. After pretreatment with coagulation-flocculation process, 76% of the COD was successfully removed. After this stage, two different NF membranes (NP010 and NP030) were tested at different pressures for further COD removal. COD removal efficiency of the NP010 and NP030 membranes increased from 78.3 to 79.3% and from 78.5 to 82.3% with increasing pressure from 10 to 30 bar, respectively (Fig. 4A). Although COD removal efficiency is high in the chemical coagulation-flocculation process, caustic loss has been the main problem. For this purpose, wastewater was filtrated using only UF membrane. COD removal efficiency of the UP150 membrane increased from 19.3 to 26.2% when pressure increased from 10 to 30 bar (Fig. 4B). The reason why the removal efficiency of the UF membrane is lower than that of the NF membrane can be explained by the fact that the pore size of the UF membrane is larger than that of the NF membranes. Thus, compounds of larger sizes pass into the filtrate from UF membranes than from NF membranes^36^. Ağtaş et al.^17^ established a pilot-scale system with a UF/NF membrane for recovering caustic wastewater used in the textile industry. They observed that caustic recovery increased at least half with each cycle. Moreover, they reported that the COD removal efficiency in each cycle was low.

#### NaOH recovery performances of the UF and NF membranes

**Fig. 5:**
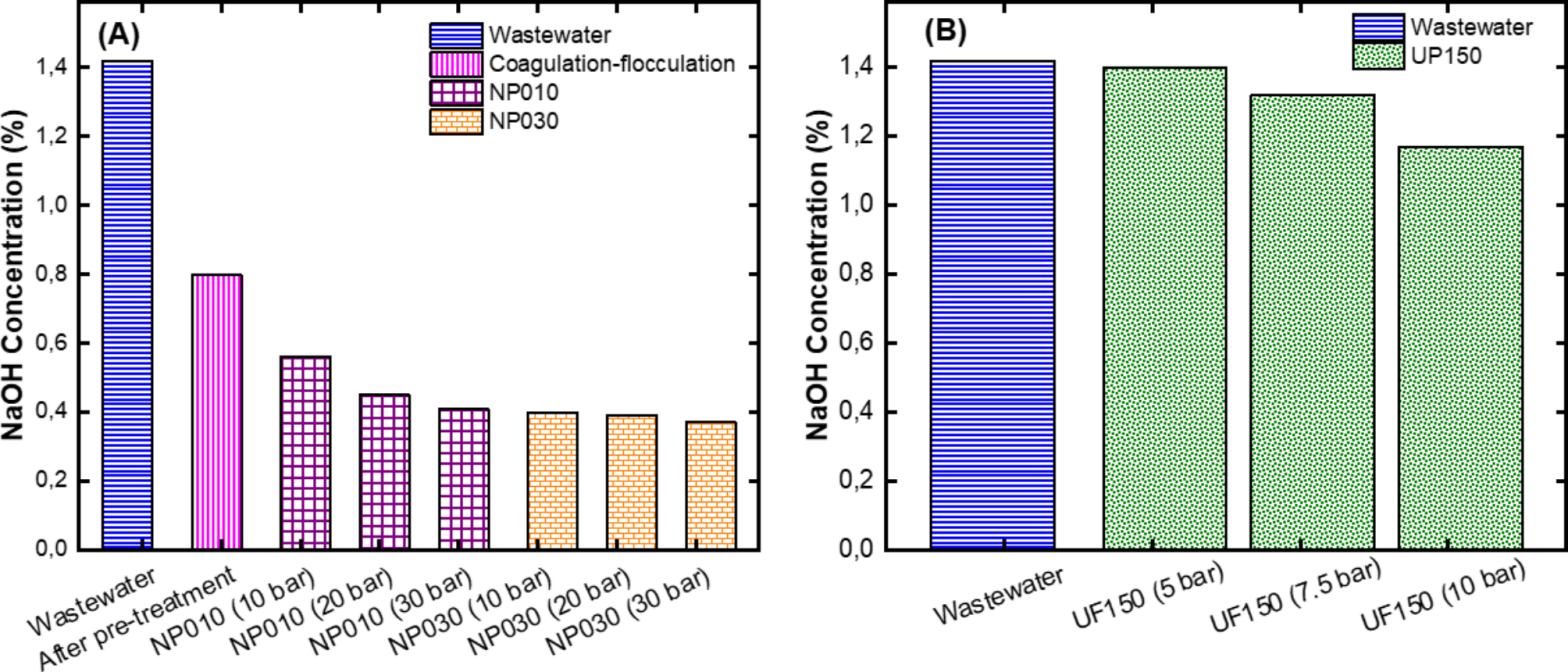
Results of NaOH concentration for **(A)** NF membranes after pre-treatment with coagulation-flocculation process and **(B)** UF membrane without pre-treatment.

NaOH is the primary source of caustic-rich wastewaters. In studies aiming to reuse or recover NaOH, the loss of it should be low. In our study, it would be appropriate to use the treatment method with the least NaOH loss among the treatment scenarios determined for this water intended to be reused in PET washing. Figure 5 shows the amount of NaOH recovered in each process. While the percentage NaOH content of the real wastewater was 1.42%, NaOH content of the pre-treated wastewater after coagulation-flocculation decreased to 0.8%. Approximately half of it was lost after coagulation-flocculation process. This situation can be attributed to the reaction between the coagulant and NaOH and its removal from the environment as sludge. Mohottige et al.^37^ examined the production of Al_2_O_3_ in wastewater with high conductivity and stated that aluminum causes the loss of organic sodium salts and caustics. After pre-treatment, NaOH concentration obtained for the NP010 (10 bar), NP010 (20 bar), and NP010 (30 bar) experimental sets were measured as 0.56%, 0.45%, and 0.41%, respectively. For the NP030 (10 bar), NP030 (20 bar), and NP030 (30 bar) membranes, this ratio was determined to be 0.40%, 0.39%, and 0.37%, respectively (Fig. 5A). The concentration of NaOH decreased after the use of the NF membranes. However, the concentration of NaOH in the filtrates obtained from the UP150 (5.0 bar), UP150 (7.5 bar), and UP150 (10 bar) membranes were measured to be 1.40%, 1.32%, and 1.17%, respectively (Fig. 5B). The amount of NaOH in the permeates collected from the NF membranes decreased with increasing pressure. This can be explained by the fact that as the amount of water passing through the membrane increased with increasing pressure^38^.

#### pH results of the UF and NF membranes

**Fig. 6:**
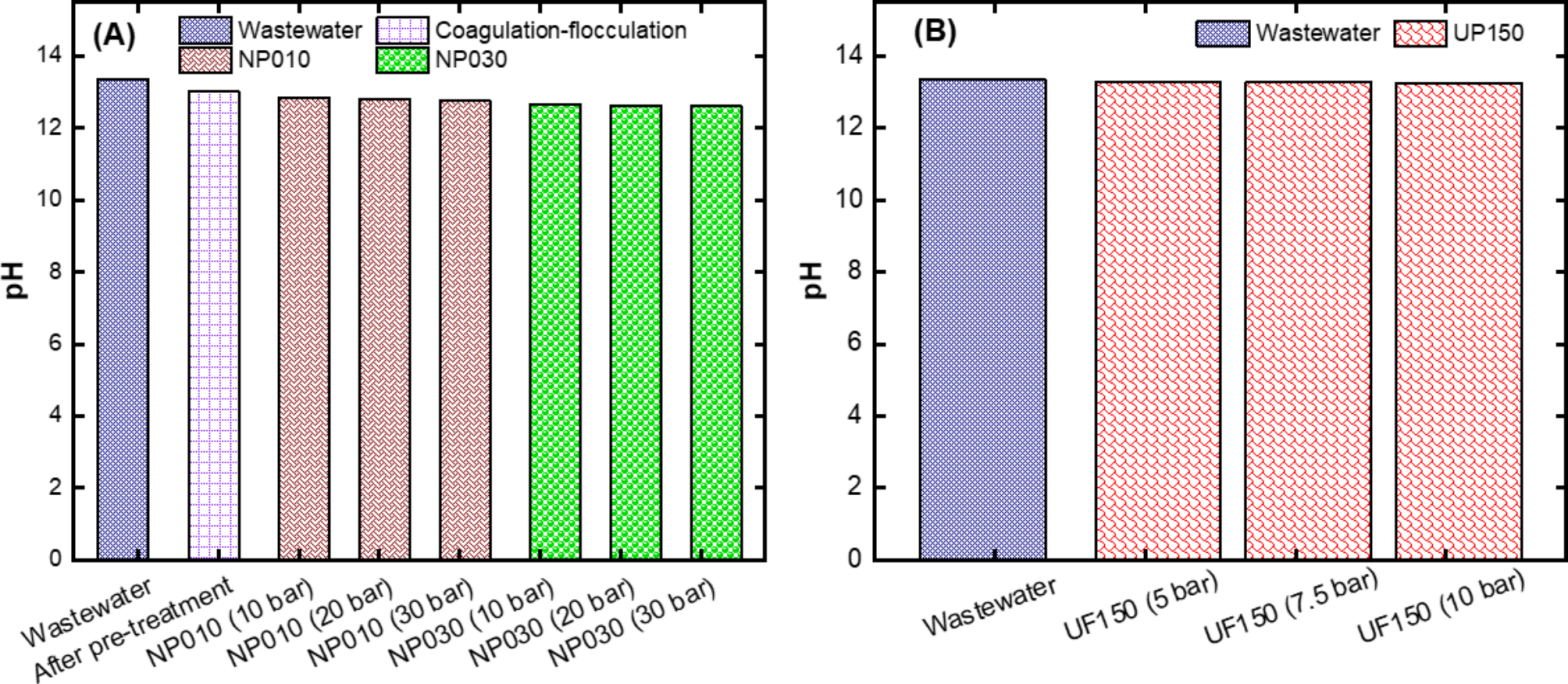
Results of pH values for **(A)** NF membranes after pre-treatment with coagulation-flocculation process and **(B)** UF membrane without pre-treatment.

It is intended that the wastewater pH level remains unchanged throughout all treatment procedures due to obtain caustic rich water. The pH of the wastewater containing NaOH is expected to be basic. The possible pH decrease in the samples obtained after treatment indicates that NaOH are removed from the water. Therefore, the pH values of the samples were measured after the treatment used in our study (Fig. 6). It was observed that the pH value in the water did not decrease as a result of all the applied treatment methods.

#### Conductivity results after membrane filtration

**Fig. 7:**
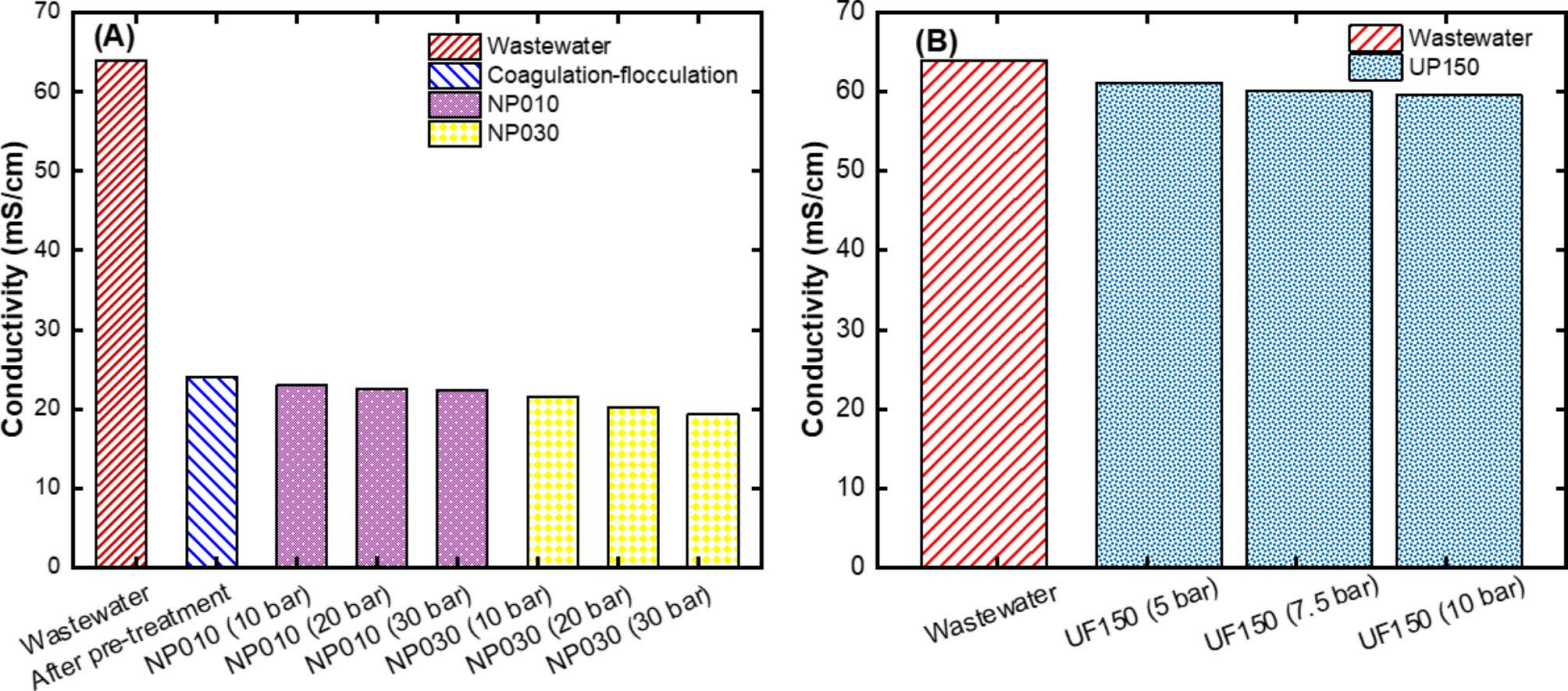
Results of conductivity values for **(A)** NF membranes after pre-treatment with coagulation-flocculation process and **(B)** UF membrane without pre-treatment.

Figure 7 shows the conductivity values in each process. The conductivity of the caustic rich wastewater used in this study was 64 mS/cm. After pre-treatment with coagulation-flocculation, this value decreased to 24 mS/cm (Fig. 7A). The removal of conductivity through coagulation-flocculation was unexpected. Conductivity reduction was also observed in a coagulation-flocculation study by Torres et al.^39^ and the conductivity of the highly conductive wastewater dropped to 72%. The similar result was reported by Mohottige et al.^37^. In their study, they examined the production of Al_2_O_3_ in wastewater with high conductivity and stated that aluminum causes the loss of organic sodium salts and caustics. The conductivity drop in our study can be explained by the possible removal of sodium ions by Al_2_O_3_ (see Fig. 5A). However, no significant decrease in conductivity was observed in the samples filtered through the UP150 membrane without pre-treatment (Fig. 7B).

**Fig. 8:**
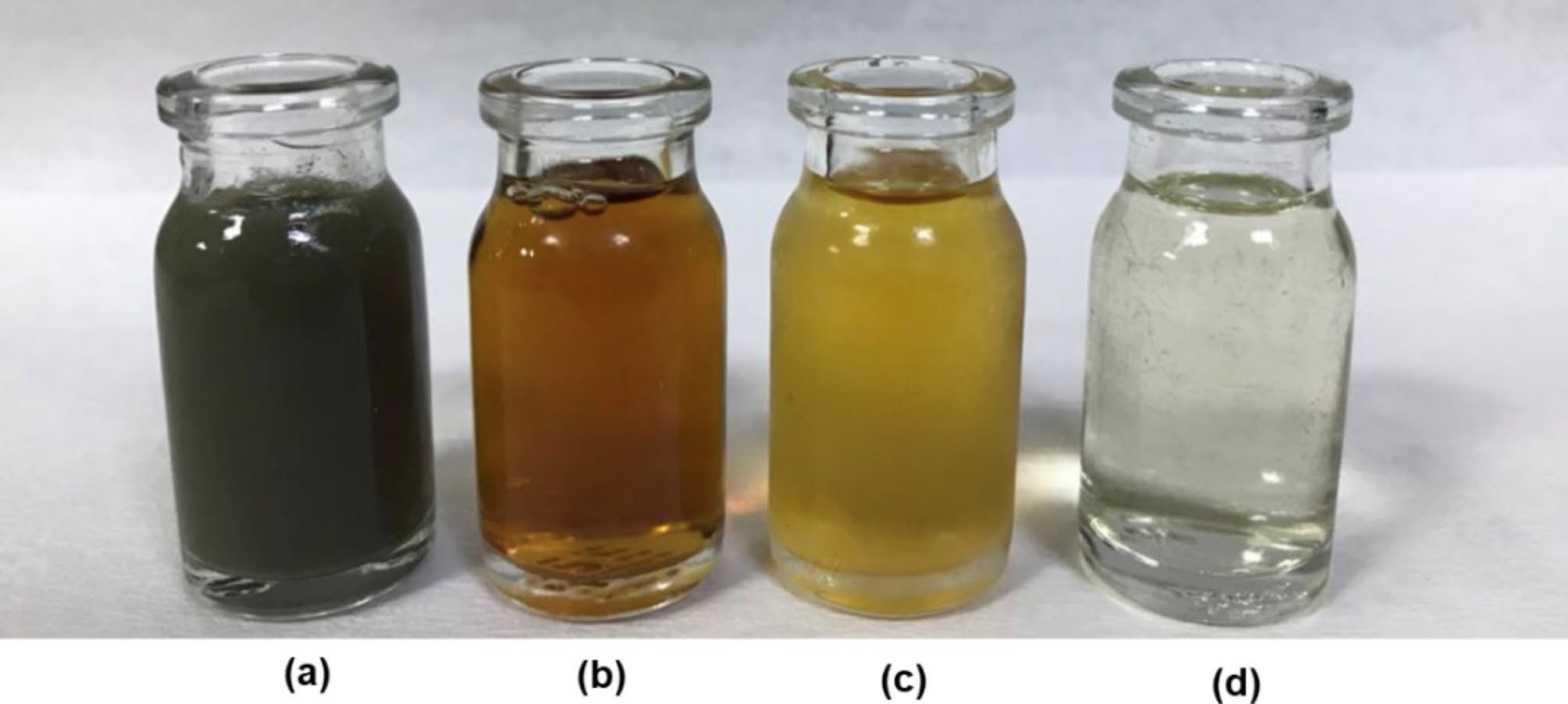
Photographs of the **(a)** raw wastewater, **(b)** after coagulation-flocculation, **(c)** NP010 effluent, and **(d)** NP030 effluent.

Photographs of the effluents obtained after each treatment method are shown in Fig. 8. Accordingly, while it was seen that the raw wastewater had a dark gray color (Fig. 8a), the color turned brown after coagulation-flocculation (Fig. 8b). The wastewater color turned to light yellow after NP010 membrane filtration (Fig. 8c) and to transparent after NP030 membrane filtration (Fig. 8d).

#### Comparison with other studies for NaOH recovery

Zhao and Xia^40^ investigated the removal of COD and recovery of sodium hydroxide from alkaline wastewater in chitin processing using stainless steel ultrafiltration membrane (SSM) and HDS-04 nanofiltration (NF) membranes. Total NaOH recovery was 98% for both membranes. They stated that SSM filtration is required for pre-treatment of alkaline wastewater before feeding into the NF system. The study conducted by Ağtaş et al.^17^ explored ceramic membrane technology for treating textile wastewater containing caustic compounds, with a particular focus on caustic chemical recovery. Their pilot-scale study evaluated three membrane configurations: ultrafiltration, nanofiltration, and combined ultrafiltration-nanofiltration system. Throughout the treatment process, they monitored multiple parameters including COD, total hardness, color, total organic carbon, sodium ion levels, pH, temperature, and membrane flux rates. Their findings demonstrated a successful NaOH recovery rate of 75%, based on the difference in sodium concentration between the membrane feed and permeate values. The researchers concluded that the permeate quality from these ceramic membrane systems showed promising potential for reuse in textile manufacturing facilities. Choe et al.^41^ investigated the recovery of caustic soda from alkaline waste of polyester fabrics using nanofiltration membranes. The flux behaviors were observed in terms of filtration time, volumetric concentration factor (VCF), operating pressure, temperature, and cleanliness. NaOH recovery was shown to be 84% with fluxes ranging from 12 to 36 L/m^2^.h. Varol et al.^42^ investigated the recovery of caustic using membrane technology from mercerized wastewater originating from a denim textile production facility. For this purpose, UF and NF membranes were used. In the first stage, flocculation, centrifugation, and microfiltration pre-treatment alternatives were evaluated in order to control possible membrane fouling, and these pre-treatment application alternatives were unsuccessful because they did not provide significant color and solid material removal. In the second stage, UF and NF processes were tested using a tight UF membrane (GR95PP) and three NF membranes (NP010, NP030, and MPF34) to recover caustic without applying any pre-treatment. As a result, they stated that they could recover approximately 99–100% NaOH from a membrane with a feed concentration of 30–40 g/L^42^.

In this study, sodium hydroxide (NaOH) recovery from alkaline wastewater formed after PET washing was investigated using UF and NF membranes. Coagulation-flocculation process was used to reduce the pollution load on the membranes before NF membrane experiments. pH, conductivity, COD removal and NaOH recovery were investigated in the effluent water as shown in Table 2. The pre-treated wastewater was filtered through NP010 and NP030 membranes in a dead-end filtration system and raw wastewater was filtered directly through a UP150 membrane. The results showed that the maximum NaOH recovery of 98.6% was obtained with the UP150 membrane at 5 bar.

**Table 2:** Characterization of raw wastewater, pretreated and filtered water from NF and UF.

Type of treatment	Parameters	pH	Conductivity (mS/cm)	COD removal efficiency (%)	NaOH recovery (%)
Pre-treatment with coagulation-flocculation process	Raw wastewater	13.35	64.0	-	-
	Al_2_**(SO**_**4**_**)**_**3**_	13.06	45.1	49.1	58.1
	FeCl_3_	12.43	58.2	32.3	63.4
	Fe_2_**SO**_**4**_	13.47	52.2	41.0	61.1
	PAC	13.18	61.3	24.2	65.4
	Al_2_**O**_**3**_	13.01	24.0	76.0	56.3
Membrane filtration process	UP150 (5.0 bar)	13.30	61.2	19.3	98.6
	UP150 (7.5 bar)	13.28	60.1	23.9	92.9
	UP150 (10 bar)	13.25	59.6	26.2	82.4
	NP010 (10 bar)	12.83	23.0	78.3	39.4
	NP010 (20 bar)	12.81	22.6	78.9	31.7
	NP010 (30 bar)	12.77	22.4	79.3	28.9
	NP030 (10 bar)	12.65	21.6	78.5	28.2
	NP030 (20 bar)	12.62	20.2	79.3	27.5
	NP030 (30 bar)	12.61	19.3	82.3	26.1

## Conclusion

Direct discharge of high pH wastewater into the environment may endanger the lives of living beings and irreparably disrupt the balance of nature. For this reason, it is extremely important to both treat such wastewater using appropriate treatment methods and apply processes to recover chemicals. In this study, as a result of coagulation-flocculation pre-treatment of PET washing wastewater, the COD removal efficiencies were determined as 49.1%, 32.3%, 41.0%, and 24.2%, respectively, for Al_2_(SO_4_)_3_, FeCl_3_, Fe_2_SO_4_, and PAC coagulants. In the coagulation process, most of the important pollutants such as suspended solids that will affect the COD value are removed. However, while the percentage NaOH content of the real wastewater was 1.42%, this value decreased to 0.8% after NaOH was precipitated as sludge due to an unavoidable reaction with the coagulant. It was determined that the UP150 membrane used under a pressure of 5 bar was suitable for NaOH recovery from PET washing wastewater. Although the pre-treatment method significantly reduced COD concentration, which can prevent the pollutant accumulation on the membrane surface, NaOH recovery was negatively affected. However, UP150 membrane was poor in terms of COD removal efficiency although it showed a good performance in NaOH recovery. Accordingly, the pre-treatment method or chemical to be used before the recovery process can be changed when NF membrane will use. Thus, wastewater containing high caustic can be recovered with membrane filtration. Considering the optimum conditions in our study, it is recommended to develop new methods to prevent clogging problems in the recovery process using UP150 membrane in pilot scale systems or to try different coagulants to prevent minimum NaOH loss.

## Data Availability

The datasets generated and/or analyzed during the current study are available from the corresponding author upon reasonable request.

## References

[1] Babuponnusami, A. et al. Advanced oxidation process (AOP) combined biological process for wastewater treatment: a review on advancements, feasibility and practicability of combined techniques. *Environ. Res.*, 116944. (2023). 10.1016/j.envres.2023.116944 37611785

[2] Razzak, S. A. et al. A comprehensive review on conventional and biological-driven heavy metals removal from industrial wastewater. *Environ. Adv.* **7**, 100168 (2022).

[3] Dey, S., Veerendra, G. T. N., Babu, P. A. & Manoj, A. P. *Evaluate the use of Flower Waste Biosorbents for Treatment of Contaminated Water* (Water-Energy Nexus, 2023).

[4] Al-Taai, S. H. H. Water pollution Its causes and effects. In IOP Conference Series: Earth and Environmental Science, 790 (1), 012026). (2021).

[5] Shahedi, A., Darban, A. K., Taghipour, F. & Jamshidi-Zanjani, A. A review on industrial wastewater treatment via electrocoagulation processes. *Curr. Opin. Electrochem.* **22**, 154–169 (2020).

[6] Niknejad, A. S., Bazgir, S., Ardjmand, M. & Shirazi, M. M. A. Spent caustic wastewater treatment using direct contact membrane distillation with electroblown styrene-acrylonitrile membrane. *Int. J. Environ. Sci. Technol.* **18**, 2283–2294 (2021).

[7] Kharraz, J. A. et al. Membrane distillation bioreactor (MDBR) for wastewater treatment, water reuse, and resource recovery: a review. *J. Water Process. Eng.* **47**, 102687 (2022).

[8] Al-Hazmi, H. E. et al. *Wastewater Treatment for Reuse in Agriculture: Prospects and Challenges*116711 (Environmental Research, 2023). 10.1016/j.envres.2023.116711 37487927

[9] Singh, R., Samal, K., Dash, R. R. & Bhunia, P. Vermifiltration as a sustainable natural treatment technology for the treatment and reuse of wastewater: a review. *J. Environ. Manage.* **247**, 140–151 (2019). 31247361 10.1016/j.jenvman.2019.06.075

[10] Ewuzie, U. et al. A review on treatment technologies for printing and dyeing wastewater (PDW). *J. Water Process. Eng.* **50**, 103273 (2022).

[11] Alipour, Z. & Azari, A. COD removal from industrial spent caustic wastewater: a review. *J. Environ. Chem. Eng.* **8** (3), 103678 (2020).

[12] Asgari, K. et al. *A Review on floc-flotation of fine Particles: Technological Aspects, Mechanisms, and Future Perspectives*1–28 (Mineral Processing and Extractive Metallurgy Review, 2023).

[13] Semerjian, L. & Ayoub, G. M. High-pH–magnesium coagulation–flocculation in wastewater treatment. *Adv. Environ. Res.* **7** (2), 389–403 (2003).

[14] Zhao, C. et al. Application of coagulation/flocculation in oily wastewater treatment: a review. *Sci. Total Environ.* **765**, 142795 (2021). 33572034 10.1016/j.scitotenv.2020.142795

[15] Jawahar, M. C., Geetha, M. & Ramamoorthi, M. Recovery and reuse of spent caustic Soda from Themercerizing Section of Textile Unit. *Int. J. Mech. Eng.* **6** (3), 1019–1028 (2021).

[16] Yadav, D., Karki, S. & Ingole, P. G. Nanofiltration (NF) membrane processing in the food industry. *Food Eng. Rev.* **14** (4), 579–595 (2022).

[17] Ağtaş, M., Yılmaz, Ö., Dilaver, M., Alp, K. & Koyuncu, İ. Pilot-scale ceramic ultrafiltration/nanofiltration membrane system application for caustic recovery and reuse in textile sector. *Environ. Sci. Pollut. Res.* **28**, 41029–41038 (2021). 10.1007/s11356-021-13588-0 33772717

[18] Ahmed, S. F. et al. Recent developments in physical, biological, chemical, and hybrid treatment techniques for removing emerging contaminants from wastewater. *J. Hazard. Mater.* **416**, 125912 (2021). 34492846 10.1016/j.jhazmat.2021.125912

[19] Gul, A., Hruza, J. & Yalcinkaya, F. Fouling and chemical cleaning of microfiltration membranes: a mini-review. *Polymers* **13** (6), 846 (2021). 33801897 10.3390/polym13060846 PMC8002060

[20] Jamrah, A., Al-Zghoul, T. M. & Darwish, M. M. *A Comprehensive Review of Combined Processes for Olive mill Wastewater Treatments*100493 (Case Studies in Chemical and Environmental Engineering, 2023).

[21] Ahmad, N. N. R., Ang, W. L., Teow, Y. H., Mohammad, A. W. & Hilal, N. Nanofiltration membrane processes for water recycling, reuse and product recovery within various industries: a review. *J. Water Process. Eng.* **45**, 102478 (2022).

[22] Braz, L. M., Aguiar, A. B. S., Rodriguez, R. P. & Sancinetti, G. P. Potential for anaerobic treatment of wastewater from pet bottle washing in a fluidized bed reactor. *J. Water Process. Eng.* **31**, 100817 (2019).

[23] Altieri, V. G. et al. Treating and reusing wastewater generated by the washing operations in the non-hazardous plastic solid waste recycling process: advanced method vs. conventional method. *J. Environ. Manage.* **284**, 112011 (2021). 33515837 10.1016/j.jenvman.2021.112011

[24] Ozbey-Unal, B., Balcik-Canbolat, C., Dizge, N. & Keskinler, B. Treatability studies on optimizing coagulant type and dosage in combined coagulation/membrane processes for table olive processing wastewater. *J. Water Process. Eng.* **26**, 301–307 (2018).

[25] Kowalska, I. & Klimonda, A. Application of nanofiltration membranes for removal of surfactants from water solutions. In E3S Web of conferences (Vol. 17, p. 00044). EDP Sciences. (2017).

[26] Baird, R. B., Eaton, A. D. & Rice, E. W. Standard Methods for the Examination of Water and Wastewater 23rd Edition, 5220 B. (2017).

[27] Harvey, D. *Modern Analytical Chemistry* (McGraw Hill, 2000).

[28] Boguniewicz-Zablocka, J., Klosok-Bazan, I., Naddeo, V. & Mozejko, C. A. Cost-effective removal of COD in the pre-treatment of wastewater from the paper industry. *Water Sci. Technol.* **81** (7), 1345–1353 (2020). 32616687 10.2166/wst.2019.328

[29] Jalal, G. et al. Efficient removal of dyes in textile effluents using aluminum-based coagulant. *Chem. Int.* **7** (3), 197–207 (2021).

[30] Tlaiaa, Y. S., Naser, Z. A. R. & Ali, A. H. Comparison between coagulation and electrocoagulation processes for the removal of reactive black dye RB-5 and COD reduction. *Desalination Water Treat.* **195**, 154–161 (2020).

[31] Imbrogno, A. & Schäfer, A. I. Comparative study of nanofiltration membrane characterization devices of different dimension and configuration (cross flow and dead end). *J. Membr. Sci.* **585**, 67–80 (2019).

[32] Cirillo, A. I., Tomaiuolo, G. & Guido, S. Microfiltration of concentrated albumin solutions and the role of processing conditions on membrane fouling. *Chem. Eng. J. Adv.* **16**, 100561 (2023).

[33] Shakiba, M., Nabavi, S. R., Emadi, H. & Faraji, M. Development of a superhydrophilic nanofiber membrane for oil/water emulsion separation via modification of polyacrylonitrile/polyaniline composite. *Polym. Adv. Technol.* **32** (3), 1301–1316 (2021).

[34] Kadhim, R. J., Al-Ani, F. H., Al-Shaeli, M., Alsalhy, Q. F. & Figoli, A. Removal of dyes using graphene oxide (GO) mixed matrix membranes. *Membranes* **10** (12), 366 (2020). 33255523 10.3390/membranes10120366 PMC7760904

[35] Ribeiro, T. et al. Magnetic Natural Coagulants for Plastic Recycling Industry Wastewater Treatability. *Water* **15** (7), 1276 (2023).

[36] Pasquet, P. L., Villain-Gambier, M., Ziegler-Devin, I., Julien-David, D. & Trébouet, D. Valorization of phenolic compounds from brewery wastewater: performances assessment of ultrafiltration and nanofiltration process with application of HPLC coupled with antioxidant analysis tool. *Chem. Eng. J.* **476**, 146696 (2023).

[37] Mohottige, T. N. W., Ginige, M. P., Kaksonen, A. H., Sarukkalige, R. & Cheng, K. Y. Integrating bioelectrochemical system with aerobic bioreactor for organics removal and caustic recovery from alkaline saline wastewater. *J. Environ. Manage.* **334**, 117422 (2023). 36801680 10.1016/j.jenvman.2023.117422

[38] Almojjly, A., Johnson, D. & Hilal, N. Investigations of the effect of pore size of ceramic membranes on the pilot-scale removal of oil from oil-water emulsion. *J. Water Process. Eng.* **31**, 100868 (2019).

[39] Torres, L. G., Belloc, C., Vaca, M., Iturbe, R. & Bandala, E. R. Coagulation-flocculation process applied to wastewaters generated in hydrocarbon-contaminated soil washing: interactions among coagulant and flocculant concentrations and pH value. *J. Environ. Sci. Health Part. A*. **44** (13), 1449–1456 (2009). 10.1080/10934520903217716 20183501

[40] Zhao, L. & Xia, W. Stainless steel membrane UF coupled with NF process for the recovery of sodium hydroxide from alkaline wastewater in chitin processing. *Desalination* **249** (2), 774–780 (2009).

[41] Choe, E. K. et al. NF process for the recovery of caustic soda and concentration of disodium terephthalate from alkaline wastewater from polyester fabrics. *Desalination* **186** (1–3), 29–37 (2005).

[42] Varol, C., Uzal, N., Dilek, F. B., Kitis, M. & Yetis, U. Recovery of caustic from mercerizing wastewaters of a denim textile mill. *Desalination Water Treat.* **53**, 3418–3426 (2015).

